# Peripheral blood mononuclear cell secretome for tissue repair

**DOI:** 10.1007/s10495-016-1292-8

**Published:** 2016-10-01

**Authors:** Lucian Beer, Michael Mildner, Mariann Gyöngyösi, Hendrik Jan Ankersmit

**Affiliations:** 1 Department of Biomedical Imaging and Image-guided Therapy, Medical University of Vienna, Vienna, Austria; 2 Christian Doppler Laboratory for Cardiac and Thoracic Diagnosis and Regeneration, Medical University of Vienna, Vienna, Austria; 3 Head FFG Project 852748 “APOSEC”, FOLAB Surgery, Medical University of Vienna, Vienna, Austria; 4 Department of Dermatology, Medical University of Vienna, Vienna, Austria; 5 Division of Cardiology, Medical University of Vienna, Vienna, Austria; 6 Department of Thoracic Surgery, Medical University Vienna, Vienna, Austria

**Keywords:** PBMC, Regenerative medicine, Tissue regeneration, Paracrine, Secretome

## Abstract

For almost two decades, cell-based therapies have been tested in modern regenerative medicine to either replace or regenerate human cells, tissues, or organs and restore normal function. Secreted paracrine factors are increasingly accepted to exert beneficial biological effects that promote tissue regeneration. These factors are called the cell secretome and include a variety of proteins, lipids, microRNAs, and extracellular vesicles, such as exosomes and microparticles. The stem cell secretome has most commonly been investigated in pre-clinical settings. However, a growing body of evidence indicates that other cell types, such as peripheral blood mononuclear cells (PBMCs), are capable of releasing significant amounts of biologically active paracrine factors that exert beneficial regenerative effects. The apoptotic PBMC secretome has been successfully used pre-clinically for the treatment of acute myocardial infarction, chronic heart failure, spinal cord injury, stroke, and wound healing. In this review we describe the benefits of choosing PBMCs instead of stem cells in regenerative medicine and characterize the factors released from apoptotic PBMCs. We also discuss pre-clinical studies with apoptotic cell-based therapies and regulatory issues that have to be considered when conducting clinical trials using cell secretome-based products. This should allow the reader to envision PBMC secretome-based therapies as alternatives to all other forms of cell-based therapies.

## Introduction

The concept of using paracrine factors in a therapeutic context in regenerative medicine can be seen as a consequence of the stem cell theory in the beginning of the twenty-first century. Here we will discuss the beginnings of stem cell research, with its promising initial results and subsequent disillusion, followed by the development of the secretome concept in regenerative medicine. We will discuss the term “secretome”, characterize the components of the cell secretome, describe its effects in pre-clinical and clinical studies, and then outline the challenges and opportunities of secretome-based therapies in regenerative medicine.

## Development of the paracrine/secretome hypothesis

The origin of modern cell-free therapies can be traced back to the late nineteenth and early twentieth centuries. The Swiss physician Paul Niehans (1882–1971) coined the term “cell therapy”. His cell therapy patients included celebrities such as Winston Churchill, Pope Pius XII, Agha Kahn, and Charles de Gaulle. Niehans extensively evaluated the effects of endogene rejuvenation via injection of xenogeneic cell suspensions derived from the endocrine glands, heart, kidney, liver, bone marrow, intestinal mucosa, and reticulo-endothelial system into the corresponding organ, studying whether normal function was restored. He also studied blood enriched with leukocytes and hypothesized that this form of treatment could be used for a myriad of degenerative diseases and cancer. Niehans used either fresh xenogeneic cells in suspension or lyophilized xenogeneic cells (vacuum freeze-dried) derived from young animals, such as sheep or animal fetuses, or human fetal cells [1]. Based on his experience with 3000 treated patients, Niehans postulated that xenogeneic cell infusion was well tolerated and not associated with any adverse reaction. Interestingly, as early as 1954, Rietschel and Pischinger articulated their reservations regarding the implantation of xenogeneic cells based on their data that xenograft transplantation induces a xenogeneic immune response, as well as the risk of transmitting zoonosis [2, 3].

Prior to Niehans, physicians experimented with stem cell-like cell types, including Alexander Maksimov in 1909 during his work on blood formation [4]. In addition, Elie Metchnikoff, and later Alexander Bogomolets, worked on the development of an adjuvant immunotherapy consisting of cytotoxic sera thought to prolong life and attenuate chronic diseases [5]. However, efficacy has not been systematically demonstrated, though these researchers were enthusiastic proponents of their own theories and treatment options. By modern standards, their results never progressed beyond an expert opinion and the publication of case reports. The lack of well-conducted studies in the field of “cell therapies” was the cause of well-argued skepticism throughout the twentieth century.

The current concept of using autologous or allogeneic stem cells for replacing or engineering injured tissues to re-establish normal biological function was re-envisioned in the beginning of the twenty-first century in the clinical setting of acute myocardial infarction (AMI) and chronic heart failure (HF). Initially, it was thought that the human heart is a post-mitotic and terminally developed organ with no cell renewal capacity, but this dogma was refuted in 2001 when enhanced myocyte cell proliferation was demonstrated following AMI [6] and then confirmed by other groups [7, 8]. Further studies revealed that a small number of extra-cardiac progenitor cells are capable of migrating into the damaged human myocardium following heart transplantation to replace damaged cardiomyocytes [9]. A variety of pre-clinical studies simultaneously showed striking results by using different sources of stem cells for the treatment of acute AMI. Human bone marrow-derived CD34-cells attenuated myocardial infarction in athymic rats (impaired immune system) when injected intravenously 48 h after vascular ligation [10]. Orlic et al. showed that intramyocardial injection of murine c-KIT+ bone marrow-derived cells in mice improves hemodynamic functions; the authors claimed that these stem cells enhanced myocardial regeneration by de novo generation of functional myocardium [11].

Based on these encouraging pre-clinical experiments, several groups rapidly transferred the “stem cell potential” to the realm of clinical application. Initial trials, such as the randomized controlled trial TOPCARE-AMI, were able to show minor improvements in left ventricular function following intracoronary injection of autologous progenitor cells [12]. However, ambiguous results were published in subsequent years without providing evidence that a single-cell-based therapy has significant effectiveness [13]. However, three recent meta-analyses generated contradictory results. Fisher et al. reported that autologous cell therapy may reduce the risk of mortality and re-hospitalization in patients with HF [14, 15]. Gyöngyösi et al. showed that intracoronary stem cell therapy following AMI provides no clinical benefits or reduction in mortality [16]. Gyöngyösi et al. performed an individual participant data (IPD)-based meta-analysis consisting of 12 randomized controlled trials with 1252 patients and concluded that neither clinical data nor left ventricular functions were significantly improved by intracoronary cell therapy following AMI [16]. These data highlight the lack of clinical efficacy of stem cell therapy in the clinical setting of AMI and dampen the enthusiasm for resource-intensive research into cell-based therapy. The controversial discussion on the efficacy of stem cell therapy is ongoing, and the current BAMI trial funded by the European Commission (ClinicalTrials.gov Identifier: NCT01569178) will provide definitive answers in a well-designed and controlled study.

How can we interpret the data generated during the last two decades in stem cell research and, more importantly, what lessons have been learned from these investigations? Given that the injection of diverse types of stem cells can promote cardiomyocyte cytoprotection/regeneration pre-clinically, it was initially thought that these stem cells incorporate into the injured organ and transdifferentiate into cardiomyocytes [11]. Later, it was shown that mesenchymal stem cells (MSCs) not only engraft and differentiate into cardiomyocytes, but also stimulate the proliferation and differentiation of endogenous cardiac stem cells [17], and allogeneic MSCs stimulate the proliferation of endothelial progenitor cells, restoring normal endothelial function [18].

In contrast, only a small percentage of injected stem cells engraft in the heart [19]. Penicka et al. reported that approximately 5 and 1 % of stem cells remain in the human myocardium 2 and 18 h after intracoronary infusion, respectively [20, 21]. The vast majority of cells were sequestered in the spleen, liver, lung, bone marrow, and lymph nodes a few hours after infusion [21, 22]. Subsequent studies could not reproduce the stem cell differentiation into cardiac cells. Specifically, c-KIT+ cardiac progenitor cells [23] were unable to differentiate into cardiomyocytes or support myocardial regeneration following myocardial infarction. Murry et al. failed to identify any transdifferentiation of hematopoietic stem cells into cardiomyocytes in 145 transplants into normal and injured adult mouse hearts [24].

If stem cells do not engraft in a biologically relevant manner or demonstrate long-term survival in injured heart [25], how can we explain the beneficial effects? Recent evidence suggests that, instead of direct cell fusion or transdifferentiation, paracrine factors released by injected cells are responsible for the beneficial effects. Gnecchi, using in vivo data, was one of the first to propose that infused stem cells may exert their biological effects via the secretion of paracrine factors in addition to cell–cell interactions. In 2005, this group reported that conditioned medium from MSCs under hypoxic stress significantly attenuated hypoxia-induced cell death among adult rat ventricular cardiomyocytes [26]. Gnecchi and Dzau demonstrated the importance of paracrine factors derived from stem cells in tissue regeneration in several studies and reviews [27–30]. Another pioneer in the field is Prof. Dr. KC Wollert, who speculated in 2005 about the role of paracrine factors from stem cells in regenerative medicine [31]. Interestingly, in 2008 the same group showed that supernatants obtained from PBMCs have only marginal differences from the stem cell secretome regarding the ability to promote cell proliferation. In addition, ProteinChip and GeneChip analysis of paracrine factors revealed minor differences [32], supporting the study of alternative mechanisms underlying nucleated stem cell therapy in the field of regenerative medicine.

## Death of the stem cell hypothesis

Cell necrosis-induced inflammatory reactions are an integral part of the pathophysiology of myocardial ischemia. These inflammatory processes augment cardiomyocyte dysfunction and ventricular remodeling. In 2005, Thum et al. proposed that immunomodulatory signals released by injected apoptotic stem cells may be responsible for the beneficial effects observed in the initial clinical trials utilizing ex vivo manipulated stem cells [33]. They drew attention to the fact that 5–25 % of all injected stem cells are apoptotic [34] and proposed that these dying cells attenuate inflammatory reactions via the induction of anti-inflammatory cytokines (e.g., TGF-β, IL-10) by macrophages. This hypothesis was supported by the observation that apoptotic neutrophils augment TGF-β, prostaglandin E2 (PGE2), and platelet activation factor expression in phagocytes, whereas inflammatory mediators, such as IL-1β, IL-8, and TNF-α were inhibited [35]. However, Thum et al. not only hypothesized that beneficial local immune reactions are induced by apoptotic stem cells, but also speculated that any other nucleated cell type undergoing programmed cell death may improve myocardial regeneration.

## Proof and extension of Thum’s apoptosis hypothesis

Thum’s hypothesis was proven by Ankersmit et al., who sought to answer two major questions using a wild-type rodent model of acute AMI: are PBMCs capable of attenuating myocardial damage when injected intravenously after coronary ligation and does the induction of apoptosis in PBMCs prior to injection alter their biological activity?

Using in vitro assays, Ankersmit et al. were able to show that gamma-irradiated apoptotic PBMCs attenuated lipopolysaccharide (LPS)-induced spillage of IL-6 and IL-1β in co-cultured monocytes and PBMCs [36]. In addition, irradiated PBMCs reduced T-cell proliferation in mixed lymphocyte reaction assays [36].

In in vivo experiments using a rodent model of acute AMI induced by ligation of the left anterior descending artery (LAD), the infusion of irradiated PBMC suspensions (apoptotic PBMC-S) reduced infarct size compared to animals receiving non-irradiated PBMCs (living PBMC-S) or cell culture medium alone. Functional parameters (ejection fraction, end-systolic and end-diastolic diameters) were improved in animals treated with apoptotic PBMC-S [36]. In line with these findings, qualitative and quantitative changes were observed in myocardial cell infiltrates 72 h after AMI in rats receiving apoptotic PBMC-S. In contrast, rats receiving non-irradiated PBMCs or control media had a mixed cellular infiltrate in the border zone (e.g., fibroblasts, neutrophils, lymphomononuclear cells, macrophages/monocytes, endothelial cells, and dystrophic cardiomyocytes), and rats treated with irradiated PBMCs had a massive infiltration of CD68+ mononuclear cells (monocytes/macrophages). These animals also had cells positive for c-KIT+ (CD117) and vascular endothelia growth factor receptor 2 (VEGF-R2) [36]. Ankersmit et al. also tested whether conditioned medium from apoptotic PMBCs may influence gene expression by fibroblasts and keratinocytes in vitro. Surprisingly, the co-incubation of human primary fibroblasts and keratinocytes with paracrine factors derived from apoptotic PBMCs induced a massive upregulation of MMP-9, VEGF, and IL-8. Thus, this study showed that apoptotic PBMC secretome exerts anti-inflammatory effects and reduce myocardial injury following AMI, and that the supernatants from these cells induce the expression of pro-angiogenic genes in vitro. Looking at the condition media data, it was obvious to us that the secretome of nucleated apoptotic PBMCs is the only explanation and easily obtainable solution in regenerative medicine.

## Apoptosis and the rise of the Phoenix signaling pathway

At first glance, the statement “Dying cells can augment cell survival” seems contradictory, but after detailed consideration has an evolutionary justification and rationale. Apoptosis (Greek: *apo* = off, away + *ptosis* = a falling) was coined in 1972 [37] to represent an actively enforced process during normal development and morphogenesis throughout a lifetime with characteristic morphological changes [38]. Apoptosis is an actively executed process and highly diverse between different tissues. To illustrate the magnitude of apoptosis as a physiological process aiming to maintain constant cellular self-renewal and homeostasis, every day 1 × 10^11^ human neutrophils undergo apoptosis [39] and every second approximately 3000 erythrocytes enter the apoptotic pathway and are degraded in the reticulo-endoplasmic system in the spleen and liver or are phagocytosed by macrophages [40]. Up to 95 % of all thymocytes descending in the thymus are negatively selected and undergo programmed cell death to remove autoimmune T-cells [41]. Even organs with low cellular turnover, such as the brain, discard 50 % of all cells during morphogenesis [42].

Defects during these physiological processes cause autoimmune diseases or macular degeneration in the eye [43]. Apoptotic lymphocytes are known to play important roles in the pathophysiology of several chronic inflammatory diseases and are elevated following implantation of left-ventricular assistance devices [44] or heart transplantation [45] and during hemodialysis [46, 47], sepsis [48–50], COPD, and asthma (reviewed in [51]). Thus, from this historic data set multiple pathological disease entities related to augmented initiation of apoptosis in PBMCs in the immune system can be deduced.

Apoptotic cells exert immunomodulatory effects either by releasing active immune mediators directly or indirectly via the modulation of phagocytes and dendritic cells [52]. The infusion of apoptotic cells attenuates systemic concentrations of inflammatory cytokines [53, 54], whereas anti-inflammatory cytokines, such as IL-10 [55–57] and/or TGF-β [53, 57], are released.

Apoptotic cells can also actively support regenerative and cytoprotective pathways. Figuratively speaking, the apoptotic cells alert surrounding cells of danger and help protect and survive by entering the process of programmed cell death in a kamikaze-like mission. Apoptotic cells release growth signals that stimulate cell proliferation and survival, such as nanovesicles [58], lipids [59], exosomes, and proteins [60].

## Pre-clinical application of nucleated apoptotic cells

Based on the immunomodulatory capacities of apoptotic cells during the last decade, several pre-clinical studies evaluated their usage for the treatment of autoimmune disease or organ transplantation (Table 1) (reviewed in [52]).

**Table 1:** Preclinical models of apoptotic cell therapy

Pathology	Experimental model	Therapeutic effect	Application	Cell number	Cell type	Apoptotic stimulus	Apoptotic stage	References
Chronic inflammatory diseases								
Type I diabetes	(NOD)	Prevention (Ag-specific)	i.v.	10^5^ weekly, 3 weeks	Beta cell line, NT1	UVB-irradiation	NS	[61]
	(NOD)	Prevention	i.v.	10^5^ weekly, 3 weeks	Splenic stroma cells	UVB-irradiation	NS	[62]
Experimental autoimmune encephalomyelitis (EAE)	MOG_35−55_-induced EAE (C57BL/6)	Prevention (day −8, −4, or −3, but no effect when infused day + 8) (Ag-specific)	i.v.	2 × 10^7^ (less effect with 6 × 10^6^)	MOG-expressing BALB/c T-cell line W3 versus secondary necrotic cells	Fas ligand	NS	[63, 64]
Arthritis	CIA (DBA/1) Passive Ab transfer model (K/BxN)	Prevention CIA (DBA/1) Passive Ab transfer Inefficient	i.v. or i.p.	2 × 10^7^ in total, 3 consecutive days	Thymocytes	Spontaneous (in culture), death by neglect	Average 43 % annexin-V^+^ and <5 % PI^+^ cells	[65]
	SCW-induced arthritis (Lewis rats)	Prevention (at time of SCW injection)	i.p.	2 × 10^8^	Thymocytes	γ-irradiation (15 Gy)	90–95 % annexin-V^+^ and 7AAD^neg^	[53]
	Methylated BSA-induced arthritis (C57BL/6)	After immunization	i.v.	3 × 10^7^, 3 consecutive days	Thymocytes	Dexamethasone or etoposide	60–80 % annexin-V^+^ and PI^neg^ cells	[56]
	Methylated BSA-induced arthritis (C57BL/6)	After immunization	i.v.	2 × 10^7^, 3 consecutive days	Dendritic cells (DCs), but not LPS-activated DCs	Etoposide	60–75 % annexin-V^+^ and 8–11 % PI^+^ cells	[66]
Colitis	DSS-induced colitis (BALB/c or C57BL/6)	Prevention (Ag-independent)	i.v.	2.5–3 × 10^7^	Thymocytes (syngeneic) Human PBMCs (xenogeneic)	γ-irradiation (4 Gy) or methyl-prednisolone	>60 % annexin-V^+^ and <5 % PI^+^ cells	[67]
Pulmonary fibrosis	Bleomycin-induced lung injury (C57BL/6)	Prevention (Ag-independent)	Intratracheal instillation	1 × 10^7^ (2 days after disease initiation); no effect later (day 6 or 13)	Human Jurkat T-cell line versus viable Jurkat cells; mouse thymocytes; human epithelial cell line (HeLa)	UV-irradiation	~70 % nuclear morphology by light microscopy	[68–70]
Acute inflammatory diseases								
Sepsis	Thioglycolate-stimulated peritoneum or LPS-stimulated lung (ICR)	Resolution of acute inflammation	i.p. endotracheal instillation	4 × 10^7^ (3 days after disease initiation) 1.8–2 × 10^7^ (36–48 h after LPS instillation)	Human Jurkat T-cell line versus viable Jurkat cells	UVB-irradiation	60–80 % morphology by light microscopy	[71]
	LPS-induced endotoxic shock (C57BL/6); cecal ligation and puncture sepsis (C57BL/6)	Increased survival in mice	i.v.	10^7^ (day 0, or 1, 3, 6 or 24 h after)	Neutrophils	Spontaneous (in culture), death by neglect	>50 % apoptotic cells	[54]
	Cecal ligation and puncture sepsis (C57BL/6)	Worse survival in mice	i.v.	5 × 10^7^ (before, day −5)	Spleen cells versus necrotic cells (protective effect)	γ-irradiation (10 Gy)	~40–100 % annexin-V^+^ and 7AAD^neg^. cells	[72]
	LPS-induced lung inflammation (C57BL/6)	Prevention of cellular infiltrates in the airway with reduced cell count in BALF	Intranasal delivery	10^6^ (with LPS or the day after)	DCs	UVB-irradiation	NS	[73, 74]
Fulminant hepatitis	LPS plus d-galactosamine-induced fulminant hepatitis (C57BL/6 or BALB/c)	Prevention (Ag-independent)	i.v.	1–3 × 10^7^ (day −3 to −7); 2 × 10^8^ has no effect	Spleen cells	UVB-irradiation	90–95 % annexin-V^+^ and PI^neg^. cells	[57]
			(p.v.)					
Contact hypersensitivity	Delayed-type hypersensitivity using TNBS (C57BL/6)	Prevention (Ag-specific; TNP-coupled cells)	i.v.	1 × 10^7^ (day −4 or −7 before antigenic challenge)	Spleen cells versus necrotic cells	γ-irradiation (3 Gy)	NS	[75, 76]
Transplantation								
Cardiac allograft								
Acute rejection	Different donor/recipient rat strain combinations	Prevention (donor-specific)	i.v.	5 × 10^7^ (day−7 before Tx)	Spleen cells versus necrotic or viable cells	UVB- or γ-irradiation (1.50 Gy)	~50 % annexin-V^+^ and <3 % PI^+^ cells	[77]
	Different donor/recipient mouse strain combinations	Treatment (donor-specific)	i.v.	10^7^ (day 7 after Tx)	Spleen cells versus necrotic cells	UVB-irradiation	90–95 % annexin-V^+^ and <5–10 % PI^neg^ cells	[78]
	Different donor/recipient rat strain combinations	Prevention (donor-specific)	i.v.	10^6^, 10^7^, or 10^8^ (7 days before Tx)	PBMCs versus monocyte depleted PBMCs and viable cells	Mitomycin or UVC-irradiation	NS	[79]
Chronic rejection	Intra-abdominal aortic transplantation (BALB/c into C57BL/6)	Prevention (donor-specific)	i.v.	10^7^ (day−7 before Tx)	Spleen cells versus necrotic or viable cells	UVB-irradiation	90–95 % annexin-V^+^ and <5–10 % PI^neg^ cells	[80]
Islet allograft	STZ-induced diabetes (BALB/c or C57BL/6 with 500–1000 islets from FVB mice)	Prevention (donor-specific)	i.v.	5 × 10^6^ or 5 × 10^7^ (day−7 before Tx)	Spleen cells	γ-irradiation (35 Gy)	70–85 % annexin-V^+^- and <10 % PI^+^/7AAD^+^ cells	[81]
	STZ-induced diabetes (BALB/c or C57BL/6 with 200–250 islets from C57BL/6 or BALB/c mice)	Prevention (donor-specific)	i.v	1.5 × 10^7^ (day−7 before Tx)	Spleen cells versus donor viable spleen cells	UVB-irradiation	>60 % annexin-V^+^ and PI^neg^.cells	[82]
Hematopoietic cell transplantation								
Hematopoietic engraftment	Different donor/recipient mouse strain combinations	Prevention (Ag-independent)	i.v.	5 × 10^6^ (day 0, the day of Tx)	Spleen cells (donor, recipient, or third party); xenogeneic [human] Jurkat T-cell line	γ− (40 Gy), UVB-irradiation, or Fas mAb; X ray irradiation (35 Gy)	70–85 % annexin-V^+^ and <10 % PI^+^/7AAD^+^ cells	[83–87]
Acute GvHD	Different donor/recipient mouse strain combinations	Prevention (donor-specific)	i.v.	5 × 10^6^ (day 0, the day of Tx)	Spleen cells	γ-irradiation (40 Gy)	70–85 % annexin-V^+^ and <10 % PI^+^/7AAD^+^ cells	[85]
Allo-Ab after graft rejection	Different donor/recipient mouse strain combinations	Prevention (Ag-independent)	i.v	5 × 10^6^ (day 0, the day of Tx)	Spleen cells (donor, recipient, third party)	γ-irradiation (40 Gy)	70–85 % annexin-V^+^ and <10 % PI^+^/7AAD^+^ cells	[84]
Myocardial infarction	Rat LAD ligation	Treatment	i.v.	After onset of ischemia	Lymphocytes	ATG	NS	[88]
	Rat LAD ligation	Treatment	i.v.	4 h before and 24 + 48 h after onset of ischemia	Lymphocytes	Anti-lymphocyte serum	NS	[89]

This table is adapted from [52]

*Ag* antigen, *ATG* anti-thymocyte globulin, *BALF* broncho-alveolar lavage fluid, *BSA* bovine serum albumin, *CIA* collagen-induced arthritis, *DC* dendritic cells, *DSS* dextran sulfate sodium, *EAE* experimental autoimmune encephalomyelitis, *GvHD* graft-versus-host disease, *i.v*. intravenous, *i.p*. intraperitoneal, *LPS* lipopolysaccharide, *mAb* monoclonal antibody, *MOG* myelin oligodendrocyte glycoprotein protein, *MOG*_*35*−*55*_, myelin oligodendrocyte glycoprotein peptide, *Neg*. negative, *NS* not specified, *OVA* ovalbumin, *PBMC* peripheral blood mononuclear cell, *p.v*. portal vein, *SCW* streptococcal cell wall, *STZ* streptozocin, *UV* ultraviolet, *UVB* ultraviolet B (280–320 nm), *TNBS* 2,4,6-trinitrobenzene sulfonic acid, *TNP* trinitrophenyl, *Tx* transplantation

Gray et al. reported that the infusion of apoptotic thymocytes attenuated the severity or prevented the development of collagen-induced arthritis in mice via the modulation of regulatory B cells and CD4+ T-cells [55]. Perruche et al. published comparable results using streptococcal cell wall-induced arthritis in rats. The intraperitoneal injection of gamma-irradiated apoptotic thymocytes at the time of immunization reduced disease severity [53]. In a murine model of methylated BSA-induced arthritis, the application of etoposide induced apoptotic dendritic cells, but LPS-activated apoptotic dendritic cells inhibited arthritis [90]. In addition, Grau et al. used an autoimmune mediated colitis model to evaluate the effect of gamma-irradiated apoptotic splenocytes or human apoptotic mononuclear cells. The authors were able to show that apoptotic cells attenuated pro-inflammatory cytokine release from macrophages and the severity of colitis [67]. Several groups have investigated the effects of injecting apoptotic cells in the transplant setting. The injection of apoptotic splenocytes has been shown to attenuate acute cardiac allograft rejection in rats [77] and mice [91] and ameliorate chronic allograft vasculopathy in mice [80]. In addition, gamma-irradiated splenocytes promote allogeneic bone marrow engraftment [83, 84, 92]. The infusion of apoptotic leukocytes 7 days before the administration of allogeneic pancreatic islets has been shown to improve transplant survival through the modulation of regulatory T-cells [81].

Similarly, Perotti et al. performed a clinical case study of the use of allogeneic gamma-irradiated cord blood mononuclear cells in a patient with critical limb ischemia, reporting improved wound closure and vascularity [93]. Holzinger et al. chose an alternative approach; they harvested autologous PBMCs from diabetic patients with venous foot ulcers and stimulated them ex vivo with phytohemagglutinin. They then applied these cell suspensions to the foot ulcers. The clinical effect was significant enhancement of granulation and epithelialization of the skin ulcers [94].

In an unrecognized citation, in 1970 Földes et al. investigated whether the injection of anti-lymphocyte serum, which induces apoptosis in PBMCs in vivo and vitro [95], is able to attenuate experimental AMI [89]. In their historic work, they were able to show that the injection of anti-lymphocyte serum immediately decreased ischemic myocardial damage and arrhythmia in experimental AMI. They attributed these effects to the immunosuppressive effects of the anti-lymphocyte serum. This therapy concept was confirmed and extended by Lichtenauer et al. [88]. Lichtenauer et al. injected the commercially available immunosuppressive agent rabbit ATG (rATG, Thymoglobulin, Genzyme, Germany) into rodents exposed to permanent LAD ligation [88]. rATG is a successfully applied drug in clinical transplant immunology that has a mechanism comparable to anti-lymphocyte serum. Experimental in vivo ATG treatment reduced the area of necrosis and improved myocardial function compared to control treatment. In vitro data confirmed that ATG induced the release of several pro-angiogenic proteins from rat and human PBMCs into the supernatant, such as CXLC8 (IL-8). Furthermore, these paracrine factors induced the down-regulation of p53 in cultured human cardiomyocytes, suggesting a direct cytoprotective effect. The authors concluded that, in vivo, ATG induces the apoptosis of lymphocytes, which release paracrine factors that reduce ventricular remodeling and improve cardiac function after experimental AMI [88]. Relevant to this hypothesis is a case report published in 2006, wherein a patient with giant cell myocarditis (GCM), a rare and fatal disorder, presented with ventricular arrhythmia and congestive HF with the primary misdiagnosis of myocardial infarction. This patient was saved by extracorporeal membrane oxygenation (ECMO) and concomitant application of rATG (Thymoglobuline, Sangstat) after histological proof of GCM. This was the first report in the literature of complete remission in this fatal disease and was the inspirational nidus and implicit validation for studying apoptotic PBMCs and conditioned media (i.e., PBMC-S) in experimental myocarditis and other indications [96].

Thus, most of these studies injected apoptotic splenocytes or thymocytes, and only two studies induced leukocyte apoptosis in vivo. The proposed mechanisms for these cell-based therapies include immunomodulatory effects induced by the clearance of apoptotic cells by phagocytes and characterized by the direct release of immunosuppressive factors (e.g., IL-10, TGF-β) [52]. Whether the injection of nucleated cells or their released factors is sufficient to induce the immunomodulatory systemic reactions has not yet been studied.

## Paradigm changes in tissue repair—validation of the PBMC secretome

**Fig. 1:**
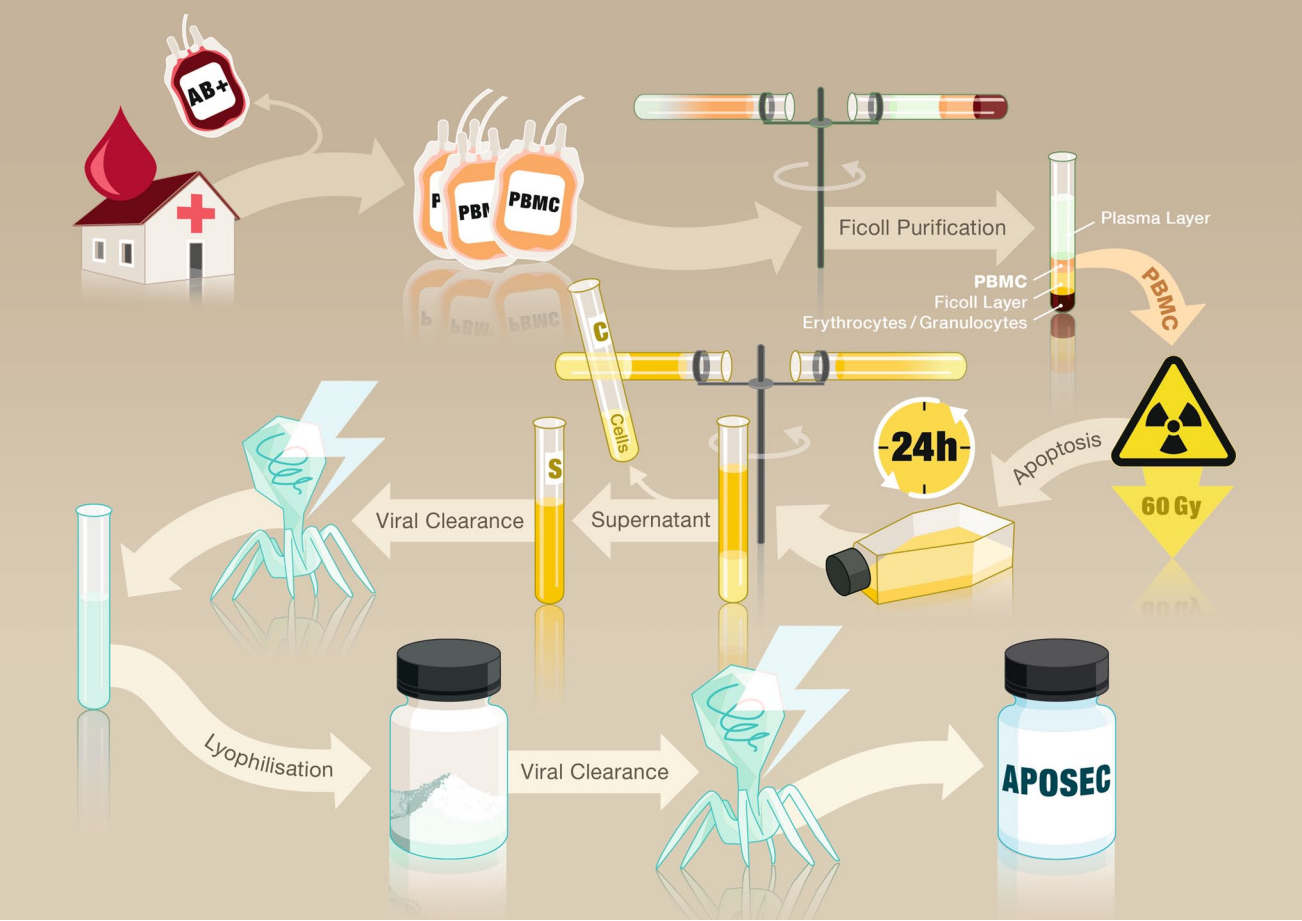
Schematic workflow of the preparation of apoptotic PBMC secretome. PBMC-enriched blood bags are obtained by blood banks and used for the generation of paracrine factors. The PBMCs are further purified using Ficoll-paque centrifugation. Apoptosis is induced by 60 Gy gamma-irradiation. The PBMCs are then cultivated at 37 °C in 5 % CO/5 % CO_2_ for 24 h. The cells are removed by centrifugation and ultrafiltration. The remaining cell culture supernatant containing the paracrine factors is subjected to methylene blue viral clearance. The viral cleared supernatant is then lyophilized to remove all soluble factors. The remaining solid compartments are subjected to a second viral clearance step and irradiated with 30k Gy, eventually yielding the final product produced in accordance with GMP

Initially, Ankersmit’s group was able to show that the injection of paracrine factors released from apoptotic PBMCs attenuates myocardial damage and preserves myocardial function following AMI in a wild type rodent model and clinically more relevant porcine large animal model [97]. Pigs receiving apoptotic cell secretome had an ejection fraction (57.0 versus 40.5 %), improved cardiac output (4.0 versus 2.4 L/min), and reduced infarct area (12.6 versus 6.9 %) compared to controls (see Table 2). Lichtenauer et al. showed that cardiomyocytes exposed to apoptotic PBMC-S increased pro-survival proteins (AKT, ERK1/2, CREB, c-Jun) and anti-apoptotic genes (Bcl-2, BAG1), and prevented starvation-induced cell death in primary cultured human cardiomyocytes. These in vitro data indicated that apoptotic PBMC-S enhances endogenous myocardial protection and repair following AMI [97]. The production process of these paracrine factors is illustrated in Fig. 1.

**Table 2:** Pre-clinical and clinical application of apoptotic PBMC-S

Species	Experimental model	Effects on disease	Application	Concentration at cultivation	PBMC source	Apoptotic stimulus	References
Rat	AMI	Reduced infarct size, improved functional parameters	i.v.	25 × 10^6^	Syngen	γ-irradiation (60 Gy)	[98]
Pig	AMI		i.v.	25 × 10^6^	Syngen	γ-irradiation (60 Gy)	[97]
Pig	AMI		i.v.	25 × 10^6^	Syngen	γ-irradiation (60 Gy)	[99]
Mice	EAM	Resolution of acute inflammation	i.p.	25 × 10^6^	Syngen	γ-irradiation (60 Gy)	[100]
Mice	Dermal wound	Improved wound healing	Topical	25 × 10^6^	Syngen	γ-irradiation (60 Gy)	[101]
Pig	Chronic HF	Improved functional parameters	i.m.	25 × 10^6^	Syngen	γ-irradiation (60 Gy)	[102]
Rat	Stroke	Reduced infarct size, improved neurological parameters	i.v.	25 × 10^6^	Syngen/human GMP viral cleared	γ-irradiation (60 Gy)	[103]
Rat	SCI	Reduced trauma size, improved neurological parameters	i.p.	25 × 10^6^	Human GMP viral cleared	γ-irradiation (60 Gy)	[104]
Pig	Dermal wound	Improved wound healing	Topical	25 × 10^6^	Human	γ-irradiation (60 Gy)	[105]
Pig	AMI	Reduced infarct size, improved functional parameters	i.v.	25 × 10^6^	Syngen GMP viral cleared	γ-irradiation (60 Gy)	[60]
Human	Dermal wound	Safety and tolerability	Topical	25 × 10^6^	Autologous GMP	γ-irradiation (60 Gy)	ClinicalTrials.gov Identifier: NCT02284360^a^

*AMI* acute myocardial infarction, *EAM* experimental autoimmune myocarditis, *HF* heart failure, *SCI* spinal cord injury, *i.v*. intravenous, *i.p*. intraperitoneal, *i.m*. intramuscular

^a^Study report—synopsis: http://www.aposcience.at/fileadmin/user_upload/MarsyasI-Clinical_study-Study_report_Synopsis.pdf

Hoetzenecker et al. showed that, in addition to an effect on intracellular myocardial signaling cascades, apoptotic PBMC-S preserved porcine epi/myocardial microvascular perfusion in a closed chest reperfusion infarction model [99]. Microvascular obstruction was measured by functional magnetic resonance imaging (fMRI) and cardiac catheterization in treated animals. Both methods revealed improved microvascular blood flow in treated animals. In addition, apoptotic PBMC-S induced up-regulation of eNOS, iNOS in cultured HUVECs, and vasodilator-stimulated phosphoprotein (VASP) activation in platelets [99]. These data indicate that apoptotic PBMC-S not only caused direct cytoprotective effects in injured hearts, but also induced systemic effects on circulating platelets and endothelial cells.

This work was extended by Pavo et al., who were able to demonstrate that apoptotic PBMC-S exerts regenerative effects in a latent porcine chronic post-myocardial infarction model [102]. In these experiments, pigs were subjected to a 90-min occlusion followed by reperfusion of the mid-left ventricular descending coronary artery. 30 days later, the animals received either apoptotic PBMC-S or control medium in the border zone after myocardial infarction via a NOGA catheter. On day 60, they were able to show that secretome-treated animals had a significantly reduced infarct size and improved cardiac index compared to controls. Transcriptional gene analysis of the myocardium revealed higher expression of protective myogenic factor Mefc2 and reduced expression of pro-apoptotic genes in treated pigs [102]. These data led the authors to conclude that apoptotic PBMC-S promotes curative gene signatures as late as 30 days after the application of PBMC-S in animals with chronic ischemic left ventricular dysfunction.

The pathophysiology and molecular processes following ischemic stroke are somewhat similar to those of AMI. Following vascular obstruction due to a ruptured atherosclerotic plaque or arterial thrombus, the downstream cells become hypoxic and eventually apoptotic/necrotic. As in myocardial infarction, immediate restoration of blood flow is the first line treatment in patients with acute ischemic stroke. Altmann et al. showed that, in a rodent model of cerebral stroke, the administration of apoptotic cell secretome reduced the ischemic area by 36 % and improved the functional neurological outcome [103]. In vitro assays revealed enhanced sprouting of human neurons and the up-regulation of pro-survival proteins (CREP, ERK1/2, c-Jun, Hsp27) in primary cultured astrocytes and Schwann cells. Interestingly, the intraperitoneal application of human apoptotic PBMC-S in naïve rodents massively increased the secretion of rat brain-derived neurotrophic factor (BDNF), a neuroprotective peptide alluded to neurogenesis. These data further support apoptotic PBMC-S having not only direct regenerative/cytoprotective effects on injured organs, but also systemic effects, overcoming tissue damage and cell death in the acute phase of hypoxia.

Similar to stroke, the neurological damage following acute spinal cord injury initiates a short initial trauma followed by a second injury caused by the endogenous immune response to the traumatic injury. This second undesirable phase of spinal cord injury consists of a multilayered and complex combination of inflammatory pathways that are thought to drive ongoing tissue damage via enzymatic degradation, oxidative stress, and blood–brain barrier dysfunction [106]. Haider et al. investigated the role of the apoptotic cell secretome in a rodent spinal cord injury model [104]. Rats treated with paracrine factors from irradiated PBMCs had significantly improved neurological function compared to the control group. Histological evaluation of spine sections revealed a smaller spinal cord cavity, reduced axonal damage, and improved vascularity index around the lesion. The PBMC secretome enhanced angiogenesis in aortic ring assays and spinal cord tissue. The authors also tested whether an immunological mechanism may be involved in neuroprotection. The secretome-treated animals exhibited enhanced recruitment of CD68-positive cells with parallel reduction in the levels of iNOS 3 days after injury. These data indicate that the PBMC secretome increases the presence of cells with known beneficial anti-inflammatory effects after spinal cord injury that accelerate the clearance of immunological disturbances, attenuating the secondary damage after spinal cord injury [104].

The PBMC secretome has been shown to enhance wound healing in vivo and in vitro [101, 105]. Mice with artificial dermal wounds were treated with emulsion containing either PBMC secretome or control medium. 6 days after injury, wound closure was significantly enhanced in secretome-treated animals compared to control animals. Histological evaluation showed accelerated re-epithelialization and neo-angiogenesis. In line with these in vivo data, experiments with keratinocytes and fibroblasts showed enhanced proliferation and up-regulation of pro-survival signaling pathways after co-incubation with PBMC secretome [101]. In a clinically relevant pig skin burn model, Hacker et al. reported that PBMC secretome accelerated wound quality, augmented neo-angiogenesis, and decreased the number of mast cells [105].

Recently, Hoetzenecker et al. investigated the capacity of apoptotic PBMC-S to modulate myocardial inflammation in a CD4-positive T-cell-dependent murine model of autoimmune myocarditis. Mice receiving apoptotic PBMC–S at the clinical onset of disease had reduced lymphocytic infiltrate, offering a novel treatment option in an experimental myocarditis model. The authors showed that PBMC-S induced caspse-8-dependent apoptosis of autoreactive CD-4-positive T-cells, leading to remission of the decorative autoimmune processes [100] in a well-defined myocarditis model.

The immunomodulatory effects of PBMC-S were further evaluated by Kasiri et al. [107]. In this study the authors were able to show that PBMC-S contains significant amounts of antimicrobial peptides. In vitro experiments revealed antimicrobial activity against Gram-negative bacteria and Gram-positive bacteria. In addition, intravenous application of apoptotic PBMC-S in rats increased de novo endogenous AMP production in vivo. The authors deduced that PBMC-S has direct and indirect positive effects on the immune system.

Based on these data, we concluded that apoptotic PBMC-S initiates multiple modes of action (MOAs) that eventually lead to cytoprotection and prevention of hypoxic tissue in vivo.

## The quest for the biologically active component(s) of apoptotic PBMC secretome

A plethora of unresolved questions are still unanswered regarding which component(s), concentration(s), and mode(s) of delivery are responsible for the effects of the PBMC secretome. A single molecule may regulate a broad spectrum of different processes, including platelet inhibition, vasodilation, neo-angiogenesis, and cytoprotection. Another option is a pleiotropy of factors alone or in combination that orchestrate different molecular cascades, leading to the beneficial effects. In order to determine the MOA of the PBMC secretome, we first have to define the secretome itself.

**Fig. 2:**
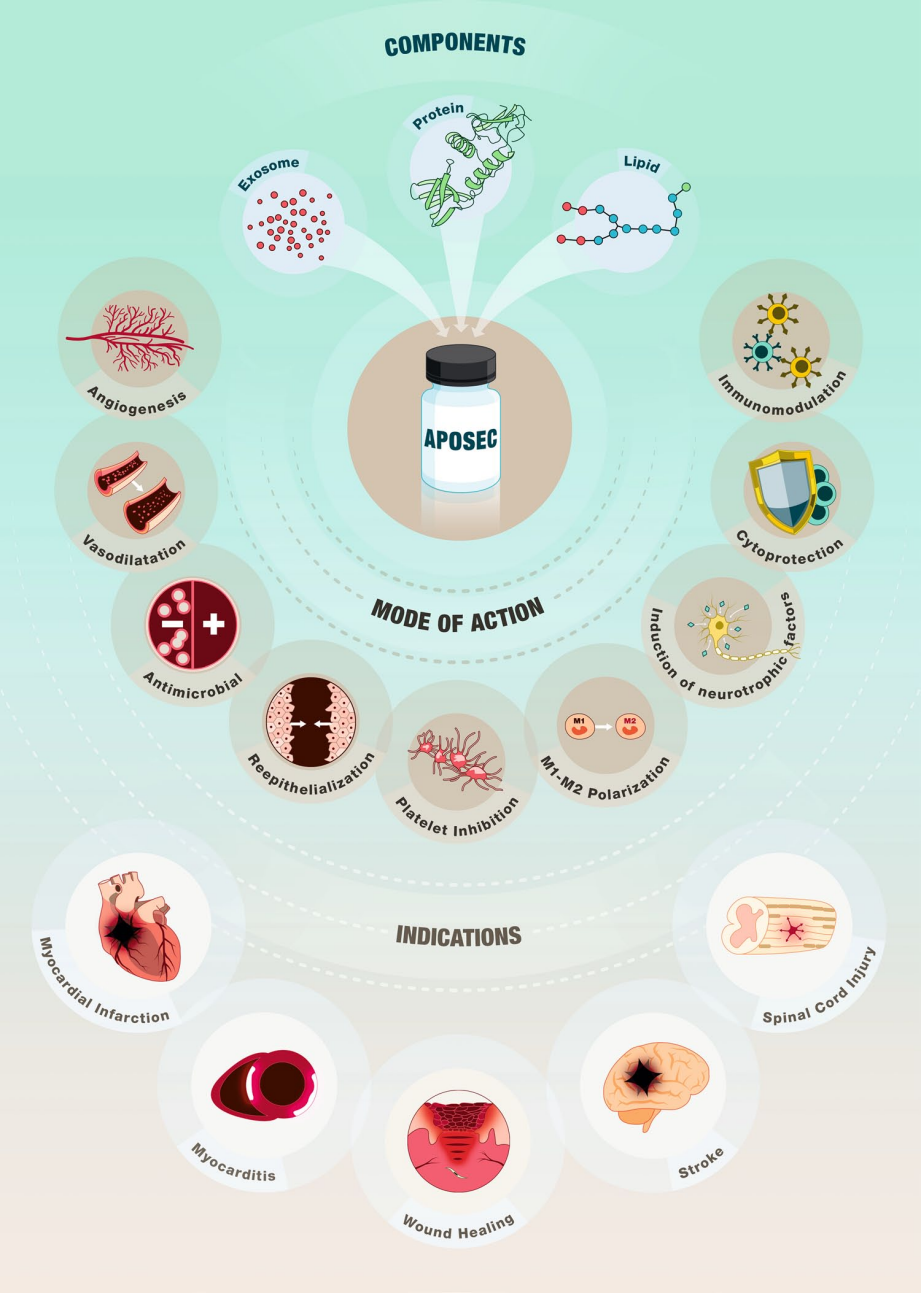
Components, mode of action, and indication of paracrine factor-based therapies. The cell secretome consists of multiple paracrine factors that can be categorized into different biological classes. The best investigated components are proteins, lipids, and exosomes, which have been shown to exhibit in vitro and in vivo biological activity. Due to the complexity of paracrine factors present in the cell secretome, it is likely that other factors exert biological activity. Paracrine factors derived from apoptotic PBMCs have been shown to induce angiogenesis and vasodilation, exert antimicrobial activity, enhance re-epithelialization, inhibit platelet coagulation, induce M1–M2 polarization, augment the release of neurotropic factors, exhibit cytoprotective capacities due to the up-regulation of anti-apoptotic proteins, and act in an immunomodulatory manner. Based on these biological effects, the PBMC secretome has been successfully tested in animal models to treat acute myocardial infarction, chronic heart failure, myocarditis, skin ulcer, stroke, and spinal cord injury

Using a systemic approach, Beer et al. tried to classify the secretome components of gamma–irradiated apoptotic PBMCs [108] based on their principle molecular characteristics in proteins (e.g., cytokines and growth factors), extracellular vesicles (e.g., microparticles and exosomes), and lipids (Fig. 2). Lichtenauer et al. performed cytokine membrane arrays using supernatant from irradiated and non-irradiated PBMCs; particularly when stressed, the supernatants contained high amounts of chemokines (e.g., CXCL8, CXCL5, CXCL1, CCL5, VEGF) [97]. We also utilized a bioinformatics-based approach in in vitro cultured PBMCs and irradiated PBMCs. A total of 213 putative actively secreted proteins were identified in the transcriptome of irradiated PBMCs and 167 transcripts in non-irradiated PBMCs [60], highlighting that irradiation causes PBMCs to become a highly active “bioreactor” while undergoing programmed cell death.

A growing body of evidence indicates that lipids released from apoptotic cells exert paracrine biological activity [109, 110]. Using the PBMC secretome, we focused on oxidized phospholipids because this group of lipids has pleiotropic biological effects ranging from the induction of endothelial cell proliferation and angiogenesis, to the attenuation of Toll-like receptor (TLR)-induced inflammation and the inhibition of oxidative burst in neutrophil granulocytes. Using high pressure lipid chromatography we measured increased concentrations of oxidized phospholipids in supernatant from irradiated PBMCs, especially PLPC-OOH, PAPC-OOH, SGPC, and PGPC [60].

In addition to proteins and lipids, the cell supernatant contains extracellular vesicles that can be isolated using an ultracentrifugation protocol, ultrafiltration, or immunoprecipitation technologies with antibody-tagged magnetic beads [111]. Extracellular vesicles are classified mainly based on the diameter, molecular weight, and surface markers. Particles with a diameter of 100–1000 nm are called microvesicles (MV) or microparticles, whereas particles with a diameter <100 nm are called exosomes (reviewed in [112]). MVs originate from plasma membrane shedding and contain high amounts of phosphatidylserine, selectin, integrin, and CD40. In contrast, exosomes are generated intracellularly and stored in the multivesicular bodies until fusion with the plasma membrane and exocytosis of the vesicles. Almost all cell types, including immune cells [113], cancer cells [114], and stem cells [115], are capable of releasing exosomes. Importantly, exosomes contain a set of proteins, lipids, messenger RNA (mRNA), and micro RNA (miRNA) that is distinct to the cytosolic compartment responsible for intercellular signaling [112].

Using both ultracentrifugation and a magnetic bead-based protocol, Beer et al. characterized exosomes and microparticles of irradiated and non-irradiated PBMCs. Gamma-irradiation induced the release of exosomes and microparticles and altered the exosomal protein content. Functional analysis revealed that exosomes and proteins are the two major biological components that induce keratinocyte and fibroblast migration and the secretion of pro-angiogenic proteins.

Although the principle components released from apoptotic PBMCs have been characterized, there are questions yet to answer. What is the biologically active component? Which factors interact with each other, whose separation would abolish the biological activity. Based on the complexity of pathological changes during an ischemic injury, it seems rather plausible to target variable pathological pathways using multifaceted drug compounds, such as apoptotic PBMC-S, instead of focusing on one single aspect. The identification of a specific cause and effect relationship on a cellular level is a matter of systems biology rather than to define a drug compound that is potentially able to treat multiple indications of unmet need.

## From bench to bedside—the potential benefit and safety of secretome-based therapy

Basic science and pre-clinical research should ultimately aim to transfer knowledge into clinical practice. Though stem cell-based clinical trials at the beginning of the twentieth century have paved the way for regenerative medicine, the first clinical trials utilizing the PBMC secretome will successfully provide proof-of-concept in humans for several indications with unmet need. Diabetic foot ulcer, AMI, stroke, and spinal cord injury are the first such indications. Ankersmit’s group recently finished a phase I study utilizing autologous GMP PBMC secretome (“Marsyas 1”, EudraCT Nr.: 2013-000756-17).

In addition to these clinically oriented issues, fundamental basic research has to be done in order to identify the biologically active components in the cell secretome and the mode of action (MOA) of these forms of therapy. For example, exosomes are currently an intensively investigated component of the cell secretome. Future research should focus on a detailed description of the composition of exosomes, including exosomal lipids, mRNAs, miRNAs, proteins, and surface receptors. We should try to understand the biological distribution of systemically injected paracrine factors to further characterize their organ-specific function.

In addition, whether apoptosis-induced lipid release has any beneficial effect in regenerative medicine is currently unknown. Oxidized phospholipids exert diverse biological functions in vitro and in vivo, suggesting that this class of lipids is responsible, at least in part, for the beneficial effect of cell secretome-based therapies.

**Fig. 3:**
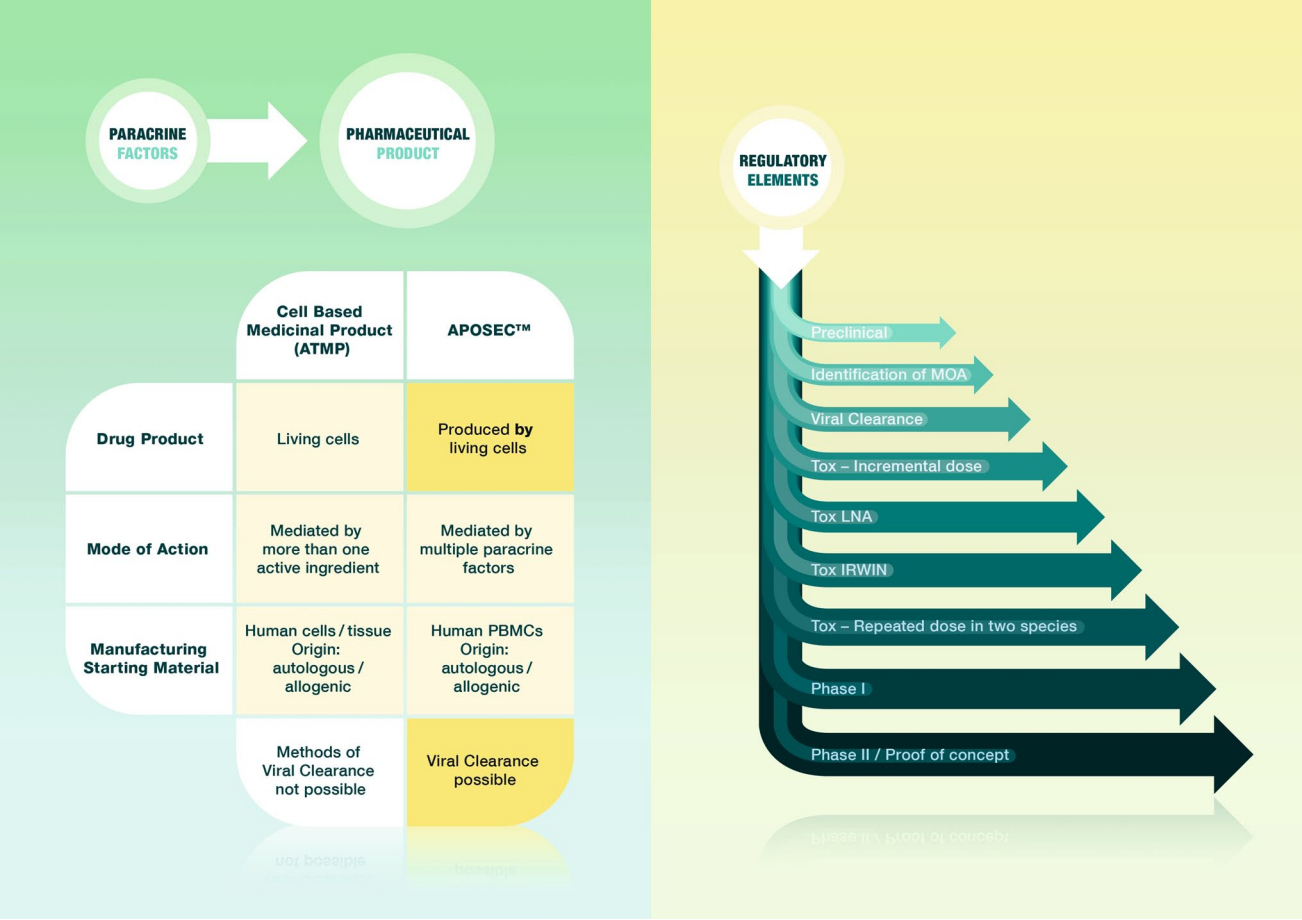
Regulatory issues for cell-free therapies. To enable regulatory approval for testing paracrine factor-based therapy, several regulatory issues have to be fulfilled. First, cell-free therapies have to be discriminated from cell-based medicinal products (ATMP). ATMPs deal with living cells, whereas paracrine factor-based therapies, such as APOSEC™, contain only factors produced by living cells. In cell therapies, the mode of action is thought to be mediated by the cells or their paracrine factors, whereas in cell-free therapies multiple paracrine factors are thought to exert the biological activity. The starting material in both therapies are cells of human origin, either autologous or allogeneic. An important difference between these two types of therapies is that viral clearance is not possible in ATMPs, but viral clearance steps can be applied in cell-free therapies such as APOSEC™. In the right side of the figure, the different regulatory steps that have to be performed during the development of a cell-free therapy are depicted, starting from pre-clinical studies and leading to Phase I/Phase II studies

Moving towards clinical application, physicians, non-clinical researchers, and investors have collaborations with reciprocal advantages for each discipline. Clinically oriented researchers and non-clinical scientists are currently in fast-moving environments that provide great opportunities for both the development and implementation of novel therapeutic modalities [116]. However, closer cooperation between the disciplines is needed to transform basic science into clinical applicability that is in line with GMP guidelines and regulatory constraints. For example, most clinically oriented scientists do not know that the regulators differentiate between so-called advanced therapy medicinal products (ATMPs) and “biologics” (Fig. 3). Paracrine-based therapies belong to the latter and are subjected to specialized regulatory restrictions if used in clinics. In Fig. 3, the different step-by-step regulatory requirements for the approval of cell-free therapies are depicted starting from extensive pre-clinical research via identification of the MOA to toxicological studies, stability assays, potency assays, and viral clearance steps, eventually resulting in clinical testing in phase I and II studies. All of these manufacturing and regulatory steps, including ethics approval and trial registration, should be completed successfully before venturing into the clinic.

In particular, knowledge of the MOA of a substance helps with regulatory approval. However, based on our experience in cell-based and paracrine research, a simplification of the monocausal effect of pleiotropic paracrine factors is considered absurd. Cell-free and cell-based therapies have multiple different molecular targets, and it is thought that only the interactions of these different effects lead to the beneficial clinical impacts observed in multiple pre-clinical studies.

Thus, a closer interaction between blood banks that know all of these regulatory issues with major pre-clinical driving forces could accelerate scientific progress.

Blood bank and transfusion units should not just follow urgent changes in clinical settings, but be actively incorporated into the process because they are familiar with GMP policy; they are familiar with handling inspections and experienced in testing biological samples [116]. Detailed knowledge of the risk involved with transfusion and transplantation, as well as avoiding microbial contamination, is crucial in setting up cell-free therapies, but these regulations are not well known by medical professionals except transfusion physicians. Blood banks could establish and run a cell-free therapy unit according to GMP. Thus, in the future, cooperation between clinically orientated scientists and blood banks could accelerate the inclusion of investors, as the certification of a validated GMP facility would result in significant (financial) risk reduction. This will eventually introduce a novel chapter in the evolution of regenerative medicine, from cell-based therapies to cell-free therapies, from stem cell therapy to white blood cell therapy, and from limited source therapy (e.g., stem cells) to an easily obtainable cell source, such as PBMCs, which are a current waste product in transfusion units.

## Conclusion

The use of human PBMCs as bioreactors for secretome-based therapies can be seen as an extension of the original concept of stem cell-based therapies to enable clinical applications. A large number of studies of transplanted stem cells have failed to show any significant integration and differentiation of injected cells into mature (cardiac) cells. In addition, there is a considerable loss of injected cells immediately after translation. Using apoptotic PBMC-S instead of stem cells, we could promote tissue repair and regeneration in multiple pre-clinical indications with the identification of a variety of MOAs. However, as stem cells are rare, their isolation and cultivation in large numbers is neither simple nor practical. Therefore, a more easily obtainable source of cells as the “bioreactor” for the secretome may facilitate the introduction of this new secretome-based medicine.

**Fig. 4:**
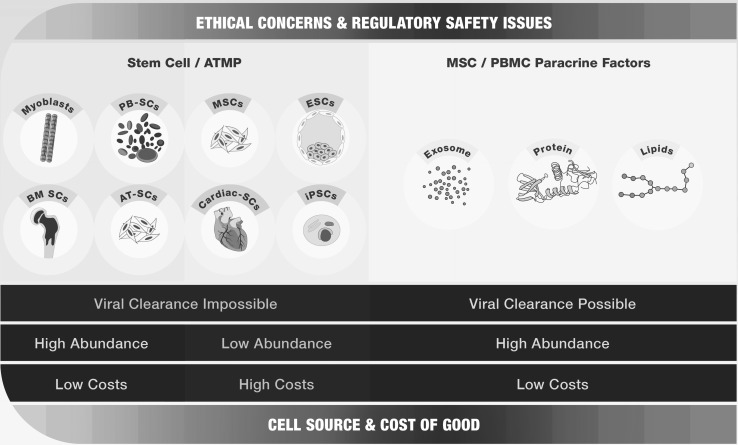
Ethical, economic, and safety considerations of stem cell-based and paracrine factor-based therapies. In contrast to stem cell-based therapies, paracrine factor-based therapies can be seen as an economic, ethical, and fully acceptable therapeutic strategy lacking significant safety issues and restrictions in production capacity. Viral clearance methods are not possible in stem cell-based therapies containing myoblasts, bone marrow stem cells (BM-SCs), peripheral blood stem cells (PB-SCs), adipose tissue-derived stem cells (AT-SCs), mesenchymal stem cells (MSCs), cardiac stem cells (Cardiac-SCs), embryonic stem cells (ESCs), and induced pluripotent stem cells (iPSCs). In addition, ESC- and iPSC-based therapies are associated with high costs for cell manufacturing and low cell numbers. Stem cell-based therapies with ESCs, and especially iPSCs, also have ethical and safety concerns because these cells have on embryonic source or bear a risk of malignant transformation. In contrast, paracrine factors derived from PBMCs or MSCs, including exosomes, proteins, and lipids, can be subjected to viral clearance methods, guaranteeing a viral-free medical drug. In addition, paracrine factors, especially from peripheral blood mononuclear cells (PBMCs), can be produced and stored in high amounts with low costs

PBMCs release pleiotropic paracrine factors that augment tissue regeneration, and their usage has the following advantages (Fig. 3): (I) PBMCs are a current waste product from each red blood donation, so their amount is unrestricted compared to any stem cell population used thus far; (II) PBMCs are obtained from mainly healthy persons, avoiding any disease- or age-dependent disadvantage in the PBMC secretome; (III) allogeneic samples with pooled PBMC factor from many individuals can be used instead of a strict autologous stem cell therapy approach; and (IV) the use of PBMCs does not comprise significant ethical problems, unlike the use of fetal stem cells (Fig. 4).

In conclusion, the PBMC secretome may potentially revolutionize regenerative medicine when appropriate proof of concept studies in humans that meet the requirements of regulating agencies are finished.
